# Association of salivary RANKL and osteoprotegerin levels with periodontal health

**DOI:** 10.1002/cre2.49

**Published:** 2017-04-12

**Authors:** A. A. Ochanji, N. K. Matu, T. K. Mulli

**Affiliations:** ^1^ University of Nairobi Kenya

**Keywords:** ELISA, osteoprotegerin, periodontal disease, RANKL

## Abstract

The aim of this study is to determine the association of salivary levels of the bone remodelling regulators (RANKL) and osteoprotegerin (OPG) with periodontal clinical status. One‐hundred fifty‐eight participants selected from a pool of adult patients via systematic random sampling were included in this descriptive cross‐sectional study carried out at the University of Nairobi Dental Hospital. About 5 ml of unstimulated whole saliva was collected from each participant followed by periodontal clinical examination. Salivary levels of RANKL and OPG were determined using human RANKL and OPG ELISA kits from R&D systems, UK. The mean salivary level of RANKL was 14.65pg/ml (+18.72 SD) with strong association to periodontal disease severity (*F* = 64.82, *p* < .001). Mean OPG levels was 139.03 pg/ml (+51.19 SD) with significantly high levels in cases without periodontitis (*F* = 19.031, *p* < .001). Consequently, a relative RANKL/OPG ratio was established with a strong positive correlation to disease severity (*r*_*s*_ = .759, *p* < .001). Statistically significant area under curve value of .932 was reported (.1613 cutoff ratio, 95% sensitivity, 6.2% specificity). The levels of RANKL and OPG in saliva and their relative ratio have strong association with the severity of periodontal disease, hence a potential adjunctive diagnostic in evaluating periodontal disease.

## CLINICAL RELEVANCE

Scientific rationale: Alveolar bone loss is a distinctive feature in periodontal diseases. RANKL induces resorption, while OPG inhibits it resulting into a relative ratio. The study focused on establishing salivary levels of the biomarkers and resolving the association of their relative ratio with periodontal health. Principal findings: Significantly higher RANKL levels and lower OPG levels were detected in severe periodontitis. Consequently, a relative RANKL/OPG ratio was established with strong positive correlation to periodontal health. Practical implications: With further clinical trials, RANKL/OPG ratio should be considered a potential adjunctive diagnostic tool and part of host response modulation in periodontal disease.

## 1 INTRODUCTION

Periodontium refers to the supporting structures of the teeth including gingival connective tissue, the periodontal ligament, the cementum, the alveolar bone, and the associated neurovasculature. Although unique in structure and location, all the components function as a single unit. Periodontal diseases affect one or more of the tissues. The National Oral Health Policy and Strategic plan of 2002–2012 (National Oral Health Policy Secretariat, 2002) recognizes that, although preventable, the prevalence of these diseases is relatively high.

Microorganisms existing in dental plaque are the primary etiological factors for the initiation of periodontal diseases. The products of these bacteria, mainly lipopolysaccharides, initiate an inflammatory response responsible for periodontal tissue destruction. The result is clinical attachment loss with apical migration of junctional epithelium and pocket formation (Kornman, 2008).

An important aspect of tissue breakdown in periodontal disease is alveolar bone loss. The disease alters the microenvironment of alveolar bone, thus compromising its structural integrity. The process is modulated by a number of molecular events including an interplay between two important biomarkers—receptor activator of nuclear factor ligand (RANKL) and osteoprotegerin (OPG; Lerner, 2004).

Receptor activator of nuclear factor ligand is a ligand that belongs to TNF family. It is expressed by osteoblasts, activated T and B cells as well as fibroblasts (Elliott, Gillespie, Horwood, Martin, & Quinn, 2000). Production is stimulated by cytokines found in the GCF/saliva as a result of inflammation in individuals with periodontal disease. The ligand binds directly to its cognate RANK receptor on the surface of preosteoclasts and osteoclasts. These results in differentiation of osteoclast progenitors and the activation of mature osteoclasts that mediates bone resorption (Seymour & Taylor, 2004).

Osteoprotegerin on the other hand is a protein that has structural homology to RANK and is therefore a decoy receptor for RANKL. The ligand (RANKL) preferentially binds to it at the expense of its natural receptor RANK. Once the interaction between the ligand and the receptor is interrupted, differentiation of osteoclasts is prevented with reduction in bone resorption (Lacey et al., 1998). A relative RANKL/OPG ratio is thus established. The ratio is significantly increased in periodontitis compared with health or gingivitis. It may therefore be a good pointer to the state of periodontal health (Kinane, Loos, & Preshaw, 2011)

Having gained significant recognition as a fluid for the detection of biological changes, saliva provides a good sample for the analysis of RANKL and OPG. Buduneli and colleagues are among the authors who have reported detectable levels of RANKL and OPG in saliva with established relationship to the clinical manifestations of periodontal disease (Biyikoglu, Buduneli, Lappin, & Sherrabeh, 2008). Collection of saliva is easy and safe. It is also noninvasive as opposed to the cumbersome and invasive conventional periodontal diagnosis. This formed the basis of the study with focus on the two biomarkers in saliva (RANKL and OPG) whose levels and ratio vary with the state of periodontal disease. The study, therefore, aimed at determining the association of salivary levels of RANKL and OPG with periodontal clinical status. The association may in future provide a platform for easy and noninvasive periodontal diagnosis. The null hypothesis was that there was no association of RANKL/OPG ratio with periodontal clinical status.

## 2 MATERIALS AND METHODS

### 2.1 Study population

This was a descriptive cross‐sectional study and was carried out at the University of Nairobi Dental Hospital between April 2015 and January 2016. Clinical data collection was planned before the index laboratory test. One‐hundred fifty‐eight participants were selected from a pool of adult patients who visited the dental hospital during the period of study via systematic random sampling method (Sample size determination is included as supplementary file for review). The study was conducted in full accordance with ethical principles, including the World Medical Association Declaration of Helsinki (version 2008). Approval was obtained from the Kenyatta National Hospital and the University of Nairobi Ethics and Research Standards Board before commencement of the study (the approval is included as a supplementary file for review). Informed and written consent was obtained from every participant prior to clinical examination and saliva sampling. Exclusion criteria included ongoing orthodontic treatment, pregnancy, and antibiotic therapy/antiseptic rinse within 3 months prior to the study or any systemic condition that could affect host response and bone metabolism (osteoporosis, rheumatoid arthritis, and diabetes).

### 2.2 Saliva sampling

About 5 ml of unstimulated whole saliva was collected from each participant. Collection was done between 7:00 and 8:00 a.m. through expectoration into a centrifuge tube (Eurotubo® Deltalab, Spain) before breakfast. There was neither a mouth rinse nor any dental procedure before collection. The samples were immediately placed in a cool box with ice pack for transportation to the lab within 1 hour for immediate centrifugation.

### 2.3 Clinical evaluation

Clinical evaluation was performed by a single calibrated examiner. The evaluation was done under illumination from dental chair light using disposable gloves, masks, gauze, a Hu‐Freidy sterile periodontal probe, and dental mirrors. The parameters assessed included gingival index based on bleeding on probing, plaque score (assessed using disclosing tablets), probing depths at six points of a tooth, gingival recession, and clinical attachment level. Periodontal disease was then classified as mild, moderate, or severe based on modification of the clinical case definitions by the Centers for Disease Control for use in population‐based surveillance of periodontitis 2007 (Eke & Page, 2007).

### 2.4 Centrifugation and storage of saliva samples

The laboratory stage of the study was performed at the Kenya AIDS Vaccine Initiative (KAVI)—Institute of Clinical Research, College of Health Sciences, University of Nairobi.

In the laboratory, each saliva sample received was assigned a serial number and recorded. The samples were immediately clarified by centrifugation for 5 min at 1,000 *g* (Heraeus Multifuge® 4KR Centrifuge).The supernatant was collected and aliquoted in 500 μL using micropipettes into clean microcap tubes (Micro tube 2 ml, PP–Sarstedt, Germany). Two aliquots were made from each saliva sample and kept in ultra‐low temperature freezer at −70°C until processing (U725 Innova® freezer, New Brunswick Scientific, last serviced by Biologic Solutions Limited in June, 2015).

### 2.5 Quantification of RANKL and OPG in saliva samples

All reagents and samples were brought to room temperature and used according to the manufacturer's instruction. Concentrations of RANKL and OPG in each sample were determined via ELISA‐based assay using commercial kits DuoSet® ELISA Development System from Bio‐Techne Corporation, R&D Systems UK (Human TRANCE/RANK L/TNFSF11, Catalog # DY626 and Human Osteoprotegerin/TNFRSF11B, Catalog # DY805). The samples were used undiluted and results expressed as picograms per milliliter (pg/ml). At the end of the assay, a stop solution was added to the ELISA plates resulting into a color change (from blue to yellow). Optical density was then determined immediately using a microplate reader with inbuilt printer (Thermo Scientific Multiskan® EX, serviced by Faram East Africa Limited in August 2015) set at 450‐nm wavelength. A standard curve was created using four*‐*parameter logistic (4PL) software by plotting the mean optical density for each standard on the *y*‐axis against respective concentration on the *x*‐axis. The formula obtained from each curve was used to derive the respective concentrations of the analytes.

### 2.6 Data entry, analysis, and presentation

The collected data was entered, cleaned, and validated. Coding and analysis was done by Statistical Packages for Social Sciences (SPSS) 20.0 for windows (SPSS Inc. Chicago, Illinois, USA) and Microsoft Excel 2013. A 4PL nonlinear regression model was used to calculate concentration of RANKL and OPG in each saliva sample. Cohen's kappa score was used to calculate both inter‐ and intra‐examiner reliability. A score of 80% was accepted. Categorical data (including gender, age groups, disease categories, and habits) were described in frequencies and percentages. Continuous data (including salivary levels of RANKL and OPG) were described using mean, range, and standard deviation (SD).

Comparison of means and proportions was done through chi square and independent *t*‐tests. Analysis of variance (anova) and Spearman's rank correlation were also used where appropriate. Independence of the association of salivary levels of RANKL, OPG, and RANKL/OPG ratio with the disease status was done through hierarchical multiple linear regression analysis, whilst adjusting for confounders such as age strata and smoking habit. Confidence level was set at 95% (α level 0.05) and presentation of findings done using tables, graphs, and box plots.

## 3 RESULTS

One‐hundred fifty‐eight participants were included in the study. Of the 158, 92 (58.2%) were females, while 66 (41.8%) were males. The age of the participants ranged between 18–75 years with a mean of 37 years (+12.74 SD). The male participants were slightly older with a mean of 38.18(+14.20 SD) compared with the female particapants with a mean of 35.64 (+11.56 SD).The difference was however not statistically significant (*t* (122) = 1.199, *p* = .233).

Oral hygiene status of participants was assessed using plaque score. The plaque score ranged between 0.58–4.33 with a mean of 2.27 + 0.77 SD showing that every participant had some degree of plaque deposits on teeth surfaces. There was statistically significant association between the plaque score and level of education (*X* ^2^ = 6.183, *p* = .045). The gingival index ranged between 0.42–2.75 with a mean of 1.56 + 0.44 SD. Association between gingival index and age was statistically significant (*X* ^2^ = 14.268, *p* = .006) with lower degree of gingival index seen in lower age groups.

The presence or absence of periodontal disease and the severity were assessed based on the consensus Centers for Disease Control/American Academy of Periodontology (AAP) definitions. Seventy‐seven participants (48.7%) had gingivitis, 39 (24.7%) had mild periodontitis, 24 (15.2%) had moderate periodontitis, while 18 (11.4%) had severe periodontitis. The association between periodontitis and age was statistically significant (*X* ^2^ = 53.845, *p* = .001)—severity of periodontitis was more in individuals in older age groups. Association with level of education was also statistically significant (*X* ^2^ = 11.416, *p* = .010).

**Figure 1:**
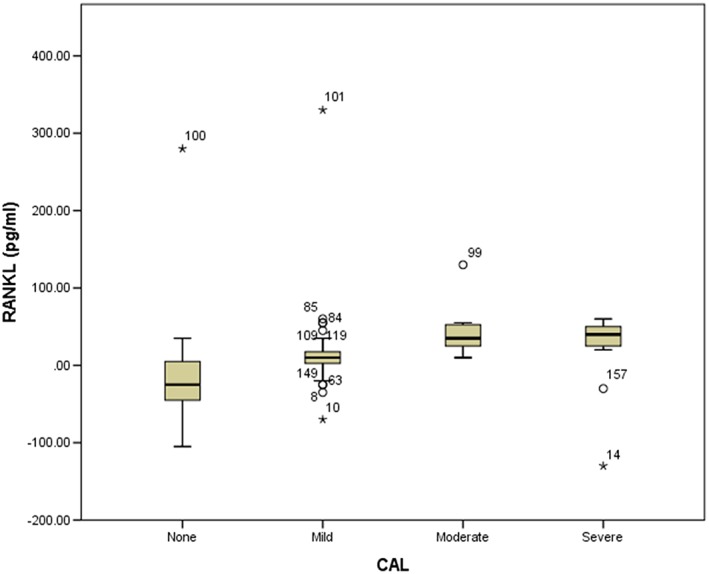
Box plot showing outlier receptor activator of nuclear factor ligand (RANKL) value

**Figure 2:**
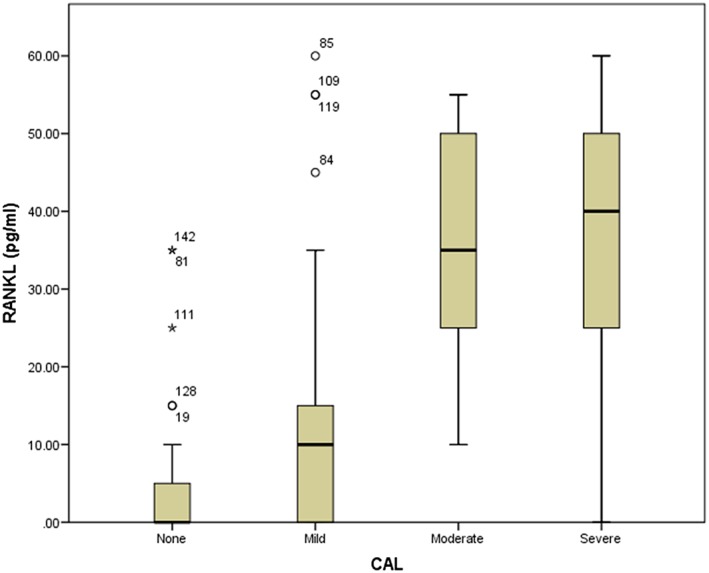
Box plot showing adjusted spread of receptor activator of nuclear factor ligand (RANKL) values

Out of the 158 samples tested, five had RANKL and OPG levels that were either too low or too high. Through the use of box plots for data distribution display (Figures 1 and 2), the five samples were deemed outliers, hence excluded from statistical analysis. Including them in the analysis would have skewed the data unfavorably. Their exclusion did not interfere with the general characteristics of parameters under study because the number recruited exceeded the calculated sample size. The negative values were undetectable, hence adjusted to zero.

The mean salivary level of of RANKL was 14.65 pg/ml (+18.72 SD) and had a strong association with the severity of periodontal disease (*F* = 64.82, *p* < .001). Higher levels were detected in severe grades of the disease. Correlations between RANKL levels and other variables are summarized in Table 1.

**Table 1:** Salivary RANKL levels by study variables

Characteristics	*n* (%)	RANKL (pg/ml)				
		(M + SD)	95% CI	df	Test	*p*‐value
Age						
18–30 years	58 (36.7)	9.20 + 15.25	5.11–13.28	2, 152	*F* = 10.377**	<.001
31–45 years	61 (38.6)	12.79 + 17.62	8.27–17.30			
>46 years	39 (24.7)	25.66 + 20.83	18.81–32.51			
Smoking status						
Nonsmokers	136 (86.1)	11.73 + 16.40	−30.79–−10.30	24.877	*t* = −4.132**	<.001
Smokers	22 (13.9)	32.27 + 22.35				
Plaque score						
1	20 (12.7)	6.25 + 11.34	0.94–11.56	2, 152	*F* = 4.979*	.008
2	77 (48.7)	12.76 + 17.21	8.83–16.70			
3	61 (38.6)	19.92 + 21.16	14.40–25.43			
Gingival index						
1	64 (40.5)	8.44 + 14.58	4.80–12.08	2, 152	*F* = 7.591*	.001
2	92 (58.2)	18.60 + 20.13	14.36–22.84			
3	2 (1.3)	37.50 + 3.54	5.73–69.27			
Periodontitis						
None	77 (48.7)	3.22 + 7.10	1.60–4.85	3, 151	*F* = 64.818**	<.001
Mild	39 (24.7)	13.42 + 16.36	8.04–18.80			
Moderate	24 (15.2)	36.96 + 14.12	30.85–43.06			
Severe	18 (11.4)	36.94 + 17.80	27.99–45.89			

*Note*. CI = confidence interval; df = degrees of freedom; M = mean; RANKL = receptor activator of nuclear factor ligand SD = standard deviation.

* *p* < .05

** *p* <.001

**Figure 3:**
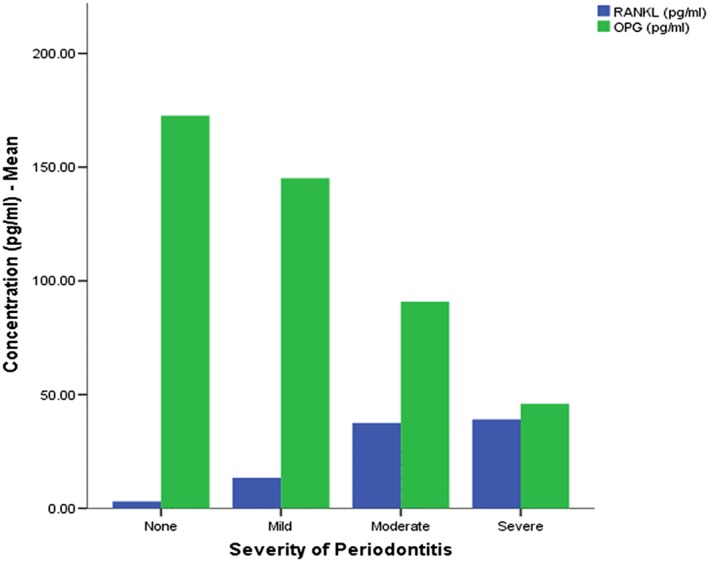
Receptor activator of nuclear factor ligand (RANKL) and osteoprotegerin (OPG) concentrations with disease category

OPG levels in the saliva samples ranged from 4.33 to 204.33 pg/ml with a mean of 139.03 pg/ml (+51.19 SD). The mean levels were significantly high in cases without periodontitis or in cases with milder grade of periodontitis (*F* = 19.031, *p* < .001). While the concentration of RANKL increased with increase in disease severity, the concentration of OPG decreased with increase in disease severity (Figure 3). As a consequence, a relative ratio (RANKL/OPG ratio) whose association with the severity of periodontitis was statistically significant got established. A Spearman's rank order correlation revealed a strong, positive correlation between the ratio and disease severity (*r*_*s*_ = .759, *p* < .001) as shown in Table 2.

**Table 2:** Spearman's rank correlation for RANKL/OPG ratio

Characteristics	*n* (%)	Ratio	
		*r*_*s*_	*p*‐value
Age			
18–30 years	58 (36.7)	.310**	<.001
31–45 years	61 (38.6)		
>46 years	39 (24.7)		
Smoking			
Nonsmokers	136 (86.1)	.308**	<.001
Smokers	22 (13.9)		
Plaque score			
1	20 (12.7)	.212*	.009
2	77 (48.7)		
3	61 (38.6)
Gingival index			
1	64 (40.5)	.274*	.001
2	92 (58.2)		
3	2 (1.3)
Periodontitis			
None	77 (48.7)	.759**	<.001
Mild	39 (24.7)		
Moderate	24 (15.2)		
Severe	18 (11.4)		

*Note*. OPG = osteoprotegerin; RANKL = receptor activator of nuclear factor ligand.

* *p* < .05

** *p* < .001

Hierarchical multiple regression elicited a statistically significant association between severity of periodontitis and RANKL/OPG ratio (β = 0.759, *t* (157) = 12.330, *p* < .001) controlling for age (β = −0.099, *t* (157) = −1.634, *p* = .104), smoking (β = 0.215, *t* (157) = 3.973, *p* < .001), and gender (β = −0.019, *t* (157) = −0.345, *p* = .730).

**Figure 4:**
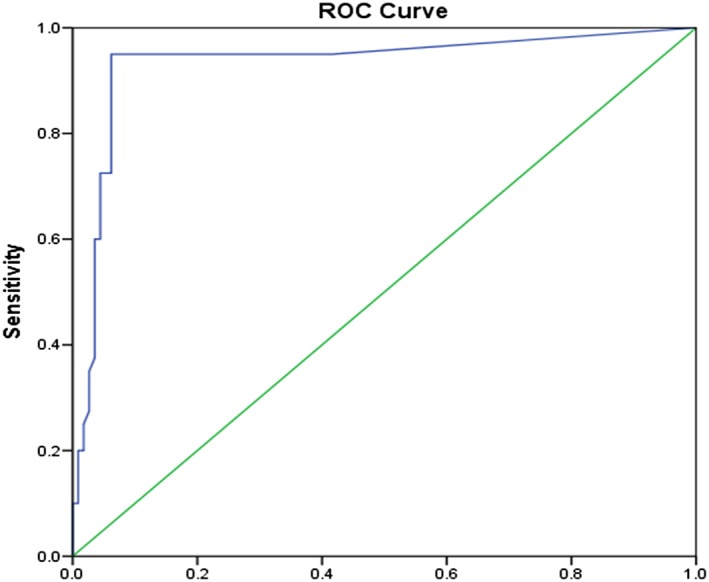
Receiver operating characteristic (ROC) curve for receptor activator of nuclear factor ligand/osteoprotegerin ratio in periodontitis

Receiver operating characteristic test was used to determine the performance of RANKL/OPG ratio as a potential diagnostic test. A statistically significant area of .932 was reported with a .1613 cutoff ratio at 95% sensitivity and 6.2% specificity levels. The test correctly identified 95% of the patients with periodontitis as true positives and 6.2% of the patients without periodontitis as true negatives at a ratio level of .1613 (Figure 4).

## 4 DISCUSSION

The salivary levels of RANKL ranged from undetectable levels to 60 pg/ml with a mean of 14.65 (+18.72 SD), while that of OPG ranged from 4.33 to 204.33 pg/ml with a mean of 139.03 (+51.19 SD). As a consequence, a relative ratio (RANKL/OPG ratio) was established with association to other parameters. The existence of the relative ratio is explained by the fact that RANKL and OPG are interrelated. They exist in inverse proportions, and their molecular interplay influence alveolar bone resorption in periodontal disease (Quinn, Horwood, Elliott, Gillespie, & Martin, 2000). Production of RANKL is stimulated by inflammatory cytokines found in body fluids such as saliva. Its presence mediates alveolar bone destruction by stimulating osteoclasts. On the other hand, OPG is the natural inhibitor of alveolar bone resorption. Being a decoy receptor, RANKL binds to it (instead of binding to RANK) preventing osteoclast differentiation (Lacey et al., 1998).

Compared with levels reported by Buduneli and colleagues in 2008 (20–200pg/ml), the RANKL levels in this study were lower (Buduneli, Biyikoglu, Sherrabeh, & Lappin, 2008). This could be attributed to difference in population sampled as well as differences in the sensitivity of instruments used. The OPG levels, however, compared well.

In this study, a statistically significant, positive correlation was found between increasing age and RANKL/OPG ratio. The correlation is in agreement with earlier studies that reported significant age‐dependent patterns in the expression of RANKL and OPG (Glowacki, Makhluf, Mizuno, & Mueller, 2000). The pattern is representative of the ageing‐cumulative characteristics of periodontal damage due to prolonged exposure to risk factors.

Another significant correlation was between RANKL/OPG ratio and smoking status—the ratio was higher in smokers than nonsmokers. Confirming the above findings, Cesar‐Neto et al. in 2007 reported that the expression of OPG gene in the gingival tissues was 1.4‐fold lower, with RANKL/OPG ratio that was 1.6‐fold higher in smokers compared to nonsmokers (César‐Neto et al., 2007). They, however, used polymerase chain reaction method and gingival tissue. Tobo'n‐Arroyave and colleagues (using the same technique as the one in this study) reported similar findings in saliva through ELISA (Tobo'n‐Arroyave et al., 2012). The altered RANKL/OPG ratio is attributed to major histopathological changes that occur in the oral cavities of smokers. The changes include altered immune response, altered vascular system, and lowered oxygen tension (Johnson & Slach, 2001). The study, within its limits, did not find strong correlation between RANKL/OPG ratio and gender.

Positive correlations were found between RANKL/OPG ratio and plaque score as well as gingival index. Similar findings were reported by Belibasakis and Bostanci in 2012 (Belibasakis & Bostanci, 2012).The correlations were however weak. The weak correlation may be attributed to limited changes in alveolar bone during gingivitis (gingival inflammation without tissue loss).

The study found a strong positive correlation between RANKL/OPG ratio and severity of periodontitis (*r*_*s*_ *=* .759, *p* < .001). Hierarchical multiple regression was used to control for the confounders (age and smoking status). Severe grade of periodontitis corresponded to higher RANKL/OPG ratio. The present findings concur with most of the previous studies (Tobo'n‐Arroyave et al., 2012, Sakellari, Menti, & Konstantinidis, 2008). Collectively, the studies indicate that the relative RANKL/OPG ratios increase as the severity of periodontitis advances. The inflammatory pathway in the periodontium explains this occurrence. As inflammation advances due to persistent microbial challenge, macrophages and dendritic cells present antigens that activate the adaptive immune system. T and B cells then accumulate and ultimately dominate the lesion. The lesion then takes a chronic course (Seymour & Taylor, 2004). Activated T and B cells are considered the major cellular sources of RANKL (Kawai, 2006). Increased production of RANKL causes an increase in the relative RANKL/OPG ratio resulting into more periodontal destruction. OPG, on the other hand, is a decoy receptor for RANKL, and its presence is protective against periodontal breakdown. RANKL binds to it (instead of binding to RANK) preventing osteoclast differentiation (Lacey et al., 1998).

Some studies have however failed to report significant correlations between RANKL/OPG ratio and severity of periodontitis. Their findings were therefore inconsistent with the findings of this study. Lu, Chen, Chang, & Kuo in 2006 failed to draw significant correlations between the ratio and clinical measurements of periodontal disease in terms of probing pocket depth, clinical attachment loss, and extent and severity of tissue breakdown (Chang, Chen, Kuo, & Lu, 2006). The failure to report significant correlations could be attributed to the differences in protocols, methods of sampling, and processing technique. Differences in study populations, sample size, and sensitivity of the assays could as well justify their findings.

In determining the performance of salivary RANKL/OPG ratio as a potential diagnostic tool, Receiver operating characteristic test reported a statistically significant area under curve value of .932 with high positive predictability (95%) for the presence of periodontitis and a low negative predictability for the absence of periodontitis (6.2%). At a ratio level of .1613, the test had high ability to correctly identify the participants with periodontitis. However, the low specificity resulted into a proportion of disease‐free participants being told of the possibility that they have the disease (false positives).The findings are in agreement with another study done to determine host‐response markers correlated with periodontal disease (Ramseier et al., 2009). Within the limits of this study, salivary RANKL/OPG ratio is therefore robust and sensitive enough to be considered for diagnostic purposes.

The study had its own limitations. It was carried out in a hospital setup. Extrapolation of the findings to the rest of the population may thus be a challenge. Being a cross‐sectional study, the snapshot timing may not have been fully representative as the study only captured the population at a single point in time. The study design also lacked the ability to make causal inference between the variables.

As a conclusion, the levels of RANKL and OPG in saliva and their relative ratio have strong association with the severity of periodontal disease and should therefore be considered as potential adjunctive diagnostic tool for evaluating periodontal disease. They should also be considered as part of host response modulation therapies for periodontal disease. However, there is a need for more salivary proteomic studies and randomized controlled trials in Kenyan setting to fully exploit the potential application in periodontal diagnosis.

The full study protocol can be accessed at the University of Nairobi Library, School of Dental sciences. Standards for reporting diagnostic accuracy studies checklist is provided as a supplementary file for review.

## CONFLICT OF INTEREST

The authors have stated explicitly that there are no conflicts of interest in connection with this article.

## Supporting information

Sample size determination
