# Supplementary figures and images for: Association of salivary RANKL and osteoprotegerin levels with periodontal health
Source: Clin Exp Dent Res. 2017 Apr 12;3(2):45–50. doi: 10.1002/cre2.49 (PMC5719813; doi:10.1002/cre2.49)

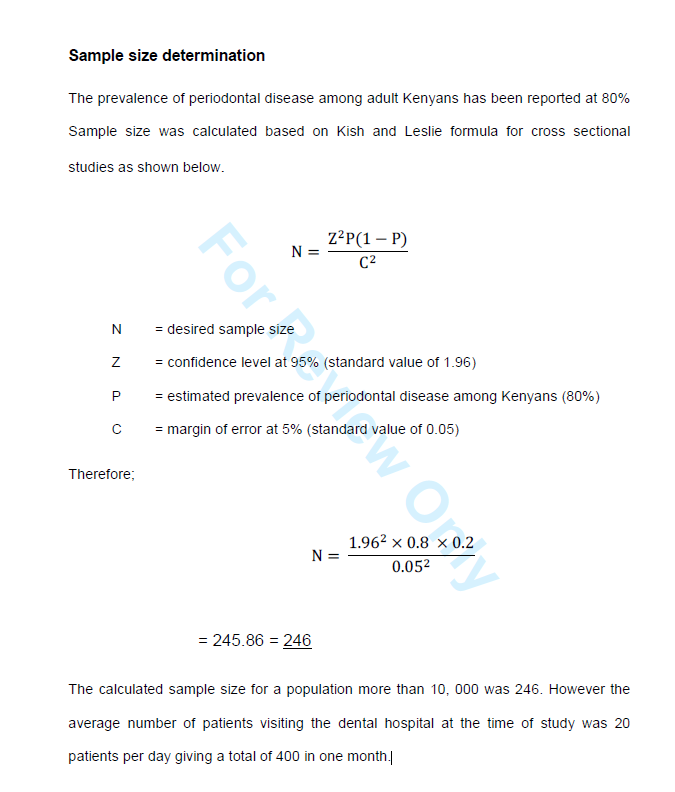


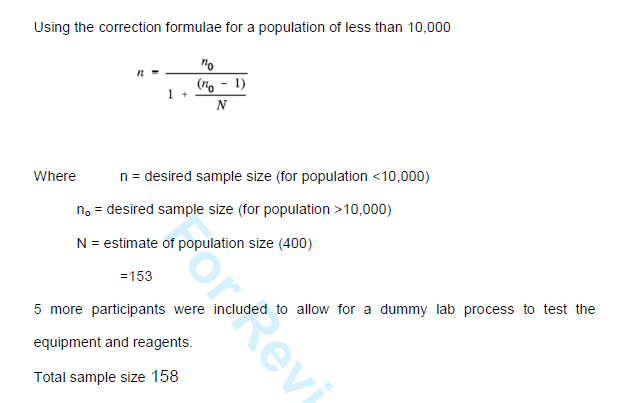

Supplement: Supplementary file 1 — Sample size determination [file CRE2-3-45-s001.docx]
